# A Case of Premature Delivery

**Published:** 1800-01

**Authors:** Thomas Denman

**Affiliations:** Old Burlington Street


### *A Case of Premature Delivery, by* Dr. Denman.

In the year 1798, a lady of rank, who was six months gone with child, came from Ireland to London, for the purpose of procuring advice; Dr. Savage and myself were called into consultation.

She had been many years married, and in her first pregnancy, through some accident, was brought to bed of a living child about the seventh month. Since that time, though she had enjoyed a good state of health, she had been delivered, after very severe labours, of four children, all of which were still-born. From the account given us, there was every reason to believe that she had been conducted with great patience and judgment through her labours; but that the pelvis was so much reduced in its dimensions, as to render it impossible for a full-grown living child to pass through it. When we had collected all the information we could, and duly weighed and deliberated upon the circumstances, we agreed in proposing to bring on her labour in the eighth month. Our opinion being represented to her friends, they having heard all the reasons we had to urge in support of it, gave their consent to the measure, though not without some hesitation; but the patient agreed to it very readily.

We fixed upon the midway, between the seventh and eighth month, as the most eligible time for the operation; and then the membranes were broken and the waters discharged. On the following day she had a rigor succeeded by fever; not in itself serious, yet which gave us some apprehension for the safety of the child; but on the third day the pains of labour came on, and she was soon delivered of a small, but apparently healthy boy. A wet nurse was immediately procured, and in the course of a few months the child became healthy and strong, and still continues to flourish. The lady recovered without interruption, and has ever since remained in perfect health.

*Old Burlington Street,*

*Nov.* 27, 1799.

THOMAS DENMAN.

